# Non-invasive modelling and parametric methods for quantification of MAO-B activity using [^11^C]L-deprenyl-D2 PET

**DOI:** 10.1177/0271678X251384264

**Published:** 2026-01-16

**Authors:** Karolina Hedman, My Jonasson, Lieuwe Appel, Andreas Tolf, Joachim Burman, Mark Lubberink

**Affiliations:** 1 Molecular Imaging and Medical Physics, Department of Surgical Sciences, Uppsala University, Uppsala, Sweden; 2 Department of Medical Physics, Uppsala University Hospital, Uppsala, Sweden; 3 Medical Imaging Centre, Uppsala University Hospital, Uppsala, Sweden; 4 Translational Neurology, Department of Medical Sciences, Uppsala University, Uppsala, Sweden; 5 Department of Neurology, Uppsala University Hospital, Uppsala, Sweden

**Keywords:** [^11^C]L-deprenyl-D2 PET, kinetic modelling, MAO-B, neuroinflammation, parametric images, reference tissue models

## Abstract

The PET radioligand [^11^C]L-deprenyl-D2 binds to monoamine oxidase B (MAO-B), enabling studies of neuroinflammation in various neurological disorders. This study aimed to evaluate non-invasive and parametric methods for quantifying [^11^C]L-deprenyl-D2 PET. Twenty-eight subjects underwent 60 min dynamic [^11^C]L-deprenyl-D2 PET. The irreversible binding parameter λ*k_3_* and the net influx rate relative to the non-displaceable volume of distribution *K_ND_* were estimated with an irreversible two-tissue plasma-input compartment model (2T3k). Non-invasive quantification of *K_ND_* was performed using cerebellum corrected for specific binding with previously suggested corrections as reference tissue. These corrections were further optimised by comparison to the 2T3k non-displaceable signal in cerebellum. Parametric images were computed using a basis function implementation of the 2T3k model, reference Patlak methods and the reduced reference tissue model. Additionally, a semi-quantitative specific binding index (SBI) was calculated as the ratio of the mean activity concentration at 30–40 or 50–60 min p.i. in target tissues to the peak cerebellum uptake. Parametric λ*k_3_* images agreed well with VOI-based nonlinear regression. Modified cerebellar reference models and SBI demonstrated poor-to-moderate correlations with arterial input-based analysis and yielded false positive differences in voxel-wise group comparisons. Full kinetic analysis with arterial input is required for accurate assessment of MAO-B activity with [^11^C]L-deprenyl-D2.

## Introduction

The radioligand [^11^C]L-deprenyl-D2 is used in positron emission tomography (PET) to study neuroinflammation. It binds irreversibly to the enzyme monoamine oxidase B (MAO-B), which is primarily located in astrocytes.^1–4^ L-deprenyl (selegiline) is a selective and irreversible inhibitor of MAO-B.^5,6^ Elevated levels of MAO-B density and activity serve as biological markers for various neurological disorders and brain abnormalities. Previous studies have demonstrated the utility of labelled L-deprenyl with PET for mapping of MAO-B in the human brain,^7–9^ and clinical PET studies have shown its application in Alzheimer’s disease,^10–14^ epilepsy,^15–17^ Creutzfeld–Jakob disease,^18^ amyotrophic lateral sclerosis,^1^ traumatic brain injury^19^ and evaluation of MAO-B inhibitors.^20–23^ This technique also holds relevance for studying multiple sclerosis (MS), a chronic inflammatory disease of the central nervous system characterised by demyelination of neuronal axons. It manifests in two forms: relapsing-remitting MS, which may eventually progress into secondary progressive MS (SPMS).^2,24–26^ In the context of MS, in vivo imaging and quantification using PET serve as a valuable tool for the diagnostic detection of reactive astrogliosis.

The gold standard method for quantifying [^11^C]L-deprenyl-D2 is the irreversible two-tissue compartment model (2T3k), which requires a metabolite-corrected plasma input function derived from arterial sampling.^8,20,27^ Although the irreversible trapping rate constant *k*_*3*_ can be considered as the primary measure of MAO-B activity, Fowler et al.^8^ proposed the macroparameter λ*k*_*3*_, defined as λ*k_3_* *=* *(K_1_/k_2_)* *k_*3*_*, as a more robust alternative to *k3* alone.^28,29^ There are several reasons for favouring λ*k*_*3*_ over *k_3_* or the more commonly used net influx rate (*K_i_* *=* *K_1_k_3_/(k_2_* *+* *k_3_)*). First, bias can occur in estimating *k_3_* and *K_i_* because estimates of *k_2_* and *k_3_* are correlated, especially in regions of high MAO-B concentrations where *k_2_* may be underestimated, leading to underestimation of *k_3_*. The issue of correlation between *k_2_* and *k*_*3*_ is mitigated by including their ratio *k*_*3*_/*k_2_*. Secondly, due the high extraction of L-deprenyl, *K_1_* is influenced by blood flow, reducing sensitivity of *K_i_* to changes in MAO-B levels. In contrast, the macroparameter λ*k*_*3*_ is, unlike *K_i_*, independent of blood flow as it incorporates the ratio *K_1_/k_2_*.^8,28–30^

Non-invasive [^11^C]L-deprenyl-D2 quantification would significantly enhance its utility by eliminating the need for arterial blood sampling and metabolite analysis, which can be both cumbersome and prone to errors. However, the widespread presence of MAO-B throughout the brain means that there is no region entirely devoid of MAO-B, and hence no suitable reference region. Therefore, two methods have previously been proposed to correct for cerebellar MAO-B binding when using a reference tissue model. One approach involves multiplying the cerebellar time-activity curve (TAC) by an exponential function,^16^ while the other utilises convolution subtraction of an exponential function.^1^ The latter method in particular has been extensively used in studies of neurodegenerative diseases involving [^11^C]L-deprenyl-D2,^11–14,31–33^ but a validation of this method by comparison to plasma-input data has never been published.

Voxel-wise quantification of [^11^C]L-deprenyl-D2 enables the generation of parametric images of MAO-B binding for visualising disease pathology. However, to the best of our knowledge, neither non-invasive methods nor voxel-wise quantification have been thoroughly validated for [^11^C]L-deprenyl-D2. Therefore, the present study aimed to evaluate and optimise non-invasive and semi-quantitative methods for kinetic analysis and to validate parametric methods for [^11^C]L-deprenyl-D2 PET. First, we will compare the previously suggested non-invasive methods and proposed improvements to these methods, including a semi-quantitative uptake measure, to plasma-input analysis. Then, we will validate parametric imaging methods by comparison to volume of interest (VOI) based methods. Finally, the limitations of non-invasive methods will be addressed by simulations assessing the effect of violations of the underlying assumptions of reference tissue models.

## Material and methods

### Subjects

Data was used from 28 subjects included in a study on MS and neuroinflammation: nine patients with SPMS, eight individuals who had undergone autologous haematopoietic stem cell transplantation (AHSCT) as a treatment for MS,^34^ and 11 healthy controls. SPMS and AHSCT patients were recruited from the Department of Neurology at Uppsala University Hospital, Sweden, while healthy controls were recruited through local and social media advertisements. The age and sex distribution was similar across the patient groups and healthy controls (mean age: 39 ± 10 years, range: 19–56 years; 19 females). The results of the evaluation of MAO-B activity within and between these groups will be presented in a separate publication. All subjects provided written informed consent prior to inclusion, and the study was conducted in accordance with the Declaration of Helsinki and approved by the Regional Board of Medical Ethics in Uppsala (EPN 2014/453/1).

### Image acquisition

Each subject underwent a 60 min dynamic PET scan following a controlled intravenous bolus injection of ~6 MBq/kg (mean 407 ± 57 MBq) [^11^C]L-deprenyl-D2^30,35,36^ (5 ml tracer solution at 1 ml/s followed by 35 ml saline at 2 ml/s). The subjects fasted for 4 h and were instructed to abstain from coffee, alcohol and tobacco for 12 h prior to the examination. The scans were performed on either an ECAT Exact HR+ (Siemens/CTI, Knoxville, TN; *n* = 12) stand-alone PET scanner with a 15.5 cm axial field of view (FOV)^37^ or a Discovery MI digital 4-ring PET/computed tomography (CT) scanner (GE Healthcare, Waukesha, WI; *n* = 16) with a 20 cm axial FOV.^38^ The dynamic PET data was corrected for attenuation either with a ^68^Ge-transmission scan (ECAT Exact HR+) or with a low-dose CT scan (Discovery MI). Images were reconstructed into 22 frames (6 × 10, 3 × 20, 2 × 30, 2 × 60, 2 × 150, 4 × 300, 3 × 600 s). Reconstruction parameters were selected to result in matching image spatial resolution of ~6.5 mm between the two scanners: ordered subsets expectation maximisation (OSEM) with six iterations, eight subsets and a 4 mm Hanning filter for the ECAT Exact HR+, and three iterations, 16 subsets and a 5 mm Hanning filter for the Discovery MI.

In addition, three-dimensional (3D) T1-weighted images were acquired on an Achieva dStream 3T magnetic resonance imaging (MRI) system (Philips Healthcare, Best, The Netherlands), using a 32-channel head coil. The T1-weighted gradient echo sequence had an acquisition duration of 3 min 50 s, a repetition time of 8.3 ms and an echo time of 3.8 ms, and a spatial resolution of 0.9 × 0.9 × 1.0 mm^3^ with 220 slices.

### Arterial blood sampling and metabolite analysis

Continuous on-line arterial blood sampling was performed during the initial 10 min of the scan (3 ml/min) using an ABSS V3 (Allogg, Mariefred, Sweden; ECAT Exact HR+) or PBS-100 (Veenstra, Joure, The Netherlands; Discovery MI). Discrete blood samples (3 ml) were drawn for metabolite analysis at 5, 10, 20, 30, 40 min p.i., and for the measurement of whole-blood and plasma radioactivity (2 ml) at 5, 10, 15, 20, 25, 30, 35, 40, 50 and 60 min p.i. Discrete blood samples were measured in a well counter that was cross-calibrated with the PET scanners. The continuous arterial sampling data were calibrated using the discrete samples taken at 5 and 10 min p.i. and extended to 60 min by a multi-exponential fit to the discrete data. The input function was then derived by multiplying the measured whole-blood data by a single exponential fit to the plasma-to-whole-blood ratios and a sigmoid function fit to the measured fraction of unmetabolised [^11^C]L-deprenyl-D2 in plasma. Finally, the input function was corrected for delay relative to the whole-brain TAC.

### Volumes of interest

Dynamic PET scans were corrected for inter-frame motion using in-house developed software in MATLAB (The MathWorks, Inc., Natick, MA). The 3D T1-weighted MR images were co-registered to the PET images using rigid transformation and segmented into grey matter, white matter and cerebrospinal fluid using SPM8 (Statistical Parametric Mapping; Wellcome Centre for Human Neuroimaging, UCL Queen Square Institute of Neurology, London, UK). An automatic probabilistic VOI template implemented in PVElab software^39^ was used to define grey matter VOIs. The data analysis included the striatum, thalamus, cerebellum and a global cortical region comprising the frontal, temporal, parietal, occipital, anterior cingulate and posterior cingulate cortex. The VOIs were overlaid onto the dynamic PET images to extract TACs.

### Tracer kinetic analysis

The irreversible two-tissue compartment model (2T3k) with plasma input function and a blood volume parameter was fitted to all TACs using weighted nonlinear regression.^27,30,40^ Parameter estimates of the model rate constants *K_1_, k_2_* and *k*_*3*_ were obtained along with the macroparameter λ*k*_*3*_,^8^ the non-displaceable volume of distribution *V_ND_* (*=* *K_1_/k_2_*), which equals λ, and the net uptake rate relative to the non-displaceable volume of distribution *K_ND_* (*=* *k_2_k_3_/(k_2_* *+* *k_3_)*). This last parameter has also been referred to as *K*, *K_i_* or *K*_i_*ref* in other studies.

Non-invasive estimation of *K_ND_* was performed using Patlak analysis^41,42^ over a time interval of 10–60 min and the reduced reference tissue model (RRTM),^43^ using reference tissue TACs as described in the next section. To further evaluate the validity of a reference tissue approach with a corrected cerebellum TAC, Spearman’s correlation, mean and standard deviation of relative differences were calculated between *V_ND_* values in the striatum and cerebellum.

Additionally, a specific binding index (SBI) was calculated as the ratio of the mean activity concentration at 30–40 or 50–60 min p.i. in target tissues to the peak value of the cerebellum TAC, providing a simplified non-invasive semi-quantitative approach without the need for kinetic modelling.

Non-invasive methods were validated by comparison to λ*k*_*3*_ values derived from the 2T3k plasma-input model using Spearman’s correlation. In addition, non-invasive *K_ND_* values were compared to 2T3k *K_ND_* using Spearman’s correlation and Deming regression.

### Reference time–activity curve

A theoretical non-displaceable reference curve, reflecting only the non-displaceable fraction of the cerebellar TAC, was calculated for each subject using the single-tissue compartment model (1T2k) and the estimated *K_1_* and *k_2_* from the 2T3k plasma-input model. Four different methods were used to correct the cerebellar TAC for MAO-B binding, of which two have been previously published (A and B):

- Multiplication of the cerebellar TAC with an exponential function, using the following equation, as previously proposed by Bergström et al.^16^:

(1) $$A:C_{\mathrm{ref}}\left(t\right)=C_{\mathrm{cer}}\left(t\right)\cdot e^{-0.04t}$$

where *C*_cer_ is the cerebellar grey matter radioactivity concentration measured at time *t* and *C*_ref_ is the cerebellar grey matter radioactivity concentration after correction for MAO-B binding.

- A modification of equation (1):

(2) $$A\prime :C_{\mathrm{ref}}\left(t\right)=\left\{\begin{array}{c} \alpha C_{\mathrm{cer}}\left(t\right)\cdot e^{-\beta t}\;\;for\;\alpha e^{-\beta t}<1\\ C_{\mathrm{cer}}\left(t\right)\;\;\;\;\;\;\;\;\;\;\;\;\;for\;\alpha e^{-\beta t}>1 \end{array}\right\}$$

Coefficients α and β were estimated by fitting the upper part of this equation to the theoretical non-displaceable reference curve for each subject for *t* > 5 min, where it follows an exponential decay, and subsequently using their median values.

- Convolution subtraction of an exponential function from the cerebellar TAC, as used by Johansson et al.^1^:

(3) $$B:C_{\mathrm{ref}}\left(t\right)=C_{\mathrm{cer}}\left(t\right)-0.01\cdot C_{\mathrm{cer}}\left(t\right)⊗e^{-0.01t}$$

- Multiplication of the cerebellar TAC with a sigmoid function:

(4) $$C:\;\;C_{\mathrm{ref}}\left(t\right)=C_{\mathrm{cer}}\left(t\right)\cdot \frac{\left(1-\gamma \cdot t^{\delta }\right)}{t^{\left(\delta +\varepsilon \right)}}$$

Here, γ,δandε were estimated by fitting equation (4) to the non-displaceable reference curve for each subject. Again, the median value of each fitting parameter for the entire dataset was subsequently used.

To assess the general applicability of these median values, differences between parameter values across groups were analysed using 95% confidence intervals (CIs). A Kruskal–Wallis test (*p* < 0.05) was performed to determine whether significant differences existed between the groups, followed by post hoc Mann–Whitney *U* tests when a significant difference was found, with Bonferroni correction applied to account for multiple comparisons.

### Parametric images

Parametric [^11^C]L-deprenyl-D2 images, showing λ*k_3_* at the voxel level, were generated using a basis function implementation^44^ of the 2T3k plasma-input model and RRTM. For this analysis, 100 basis functions were predefined, with a discrete spectrum of parameter values for the exponential clearance rate constants *k_2_* + *k_3_* ranging between 0.015 and 1 min^–1^. Furthermore, *K_ND_* was estimated at the voxel level using the reference Patlak method with the various corrected cerebellar TACs as reference at the 10–60 min time interval. SBI images were generated as described above. Parametric methods were validated by comparison of regionally averaged λ*k_3_* and *K_ND_* values from the parametric images to VOI-based λ*k_3_* and *K_ND_* values using Spearman’s correlation and Deming regression.

A voxel-wise comparison between the MS groups (SPMS and AHSCT) and the healthy controls was performed using SPM12. First, all parametric images were smoothed with an 8 mm Gaussian filter and resampled into 2 mm isotropic voxels. Then, the co-registered 3D T1-weighted MR images were normalised to Montreal Neurological Institute (MNI) standard space, and the resulting transformation matrices were applied to respective parametric images. Mean parametric images of each group were compared using an independent two-sample *t*-test (*t-*maps were thresholded at clusters >1 cm^3^, and *p* < 0.01 (uncorrected) was considered significant).

### Simulations

Simulations were performed to assess the identifiability of 2T3k *K_ND_* and the implication of violation of the assumption of equal *V_ND_* in target and reference tissues. Target and reference (without specific binding) TACs were simulated using 2T3k and 1T2k models, respectively, with a plasma input function based on the blood data of an arbitrarily chosen subject. A total of 100 TACs were generated, with rate constants randomly sampled from ranges derived from clinical data (*K_1_* = 0.4–0.7 ml.cm^–3^.min^–1^, *k_2_* = 0.1–0.2 min^–1^, *k_3_* = 0.03–0.1 min^–1^). The reference tissue *K_1_* was randomly distributed within ±0.1 ml.cm^–3^.min^–1^ of the target tissue *K_1_*. Gaussian noise was added with a noise level mimicking typical levels in both reference and target tissues. Two scenarios were simulated: (1) *k_2_* was set to result in equal *V_ND_* in reference and target tissues and (2) with differences between target and reference tissue *V_ND_* by randomly varying reference tissue *K_1_* and *k_2_* within ±15% of the values of scenario 1.

### Statistics

Statistical analysis was done as described in each section above. All kinetic modelling, parametric image generation and simulations were performed with in-house written software in MATLAB. Statistical analysis was performed in MATLAB and GraphPad Prism 10 (GraphPad Software, Boston, MA).

## Results

**Figure 1.**
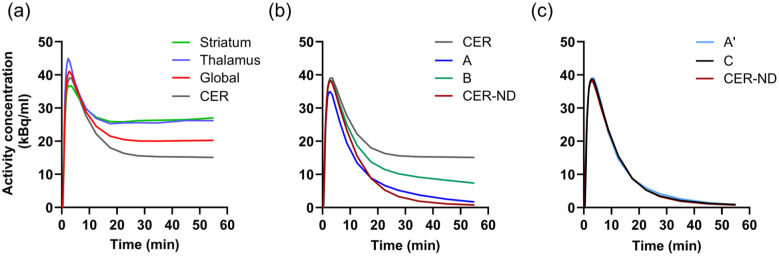
Time-activity curves for a representative subject; (a) striatum, thalamus, a global cortical region and CER; (b) uncorrected cerebellar TAC and modified cerebellar TACs using the previously suggested correction methods (A, B); (c) modified cerebellar TACs correction according to methods proposed in the current work (A′, C). The theoretical reference curve representing the CER-ND is shown in both (b) and (c). CER: cerebellum; CER-ND: non-displaceable signal in the cerebellum.

Figure 1(a) shows representative TACs in striatum, thalamus, global cortical region and cerebellum. Figure 1(b) and (c) show the different modifications of the cerebellar TAC alongside the unmodified cerebellar curve and the theoretical non-displaceable reference curve based on *K_1_* and *k_2_* of the 2T3k plasma-input model. For modification A′, based on equation (2), the median values of the coefficients were: α = 1.39 (95% CI 1.32–1.46) and β = 0.059 (95% CI 0.058–0.063). For modification C, based on equation (4), the fitted parameters differed significantly between the AHSCT group and the groups with SPMS patients and healthy controls. Nevertheless, for consistency, median values across all subjects were used: γ = 0.98 (95% CI 0.97–0.99), δ = 2.72 (95% CI 2.64–2.82) and ε = 2136 (95% CI 1482–3907).

**Figure 2.**
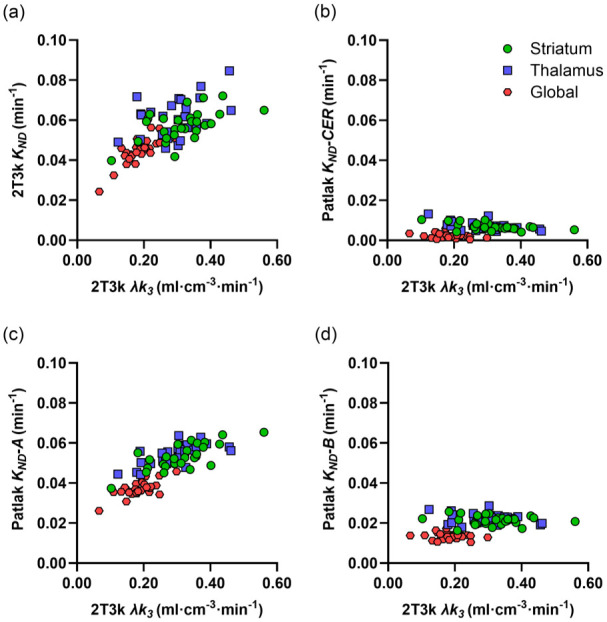
Plasma-input 2T3k λ*k_3_* values in striatum, thalamus and a global cortical region versus *K_ND_* values based on plasma-input 2T3k (a), Patlak analysis with the uncorrected CER TAC as reference (b), and Patlak analysis with modified CER reference TACs according to previously published methods A (c) and B (d). CER: cerebellum.

Figure 2 shows the relationship between VOI-based λ*k_3_* and *K_ND_* values estimated with the 2T3k plasma-input model and reference Patlak using the uncorrected cerebellum TAC, as well as the previously proposed cerebellar TAC corrections A and B. For 2T3k *K_ND_*, a moderate correlation with λ*k_3_* was obtained in the striatum and thalamus, while a stronger correlation was observed in the global cortical region. Reference Patlak *K_ND_* values, based on the corrected cerebellum TACs according to method A, showed a similar relation to λ*k_3_* as 2T3k *K_ND_*, but with higher correlations in subcortical regions than in the global cortical region. In contrast, reference Patlak *K_ND_* values estimated with method B exhibited very poor correlation with λ*k_3_*, as seen with the uncorrected cerebellum TAC. Spearman correlation coefficients of reference Patlak *K_ND_* values versus λ*k_3_* are shown in Table 1. *V_ND_* values in striatum and cerebellum, calculated using the 2T3k plasma-input model, showed a moderate correlation (ρ = 0.86; 95% CI 0.71–0.93) and a mean relative difference of 1% ± 11%.

**Table 1.** Spearman correlation (ρ) between λ*k_3_* estimates from plasma-input 2T3k and *K_ND_* estimates from non-invasive methods with different cerebellar corrections, as well as SBI for the different time intervals. Data in parentheses represent 95% confidence intervals. The significance level was <0.0001 for overall correlations, except for the correlation between 2T3k λ*k_3_* and reference Patlak *K_ND_* without any correction of the cerebellar TAC (*p* = 0.0066).

Model	Striatum	Thalamus	Global	Overall
2T3k *K_ND_*	0.49 (0.14–0.74)	0.35 (−0.04–0.65)	0.74 (0.49–0.87)	0.69 (0.56–0.79)
Patlak *K_ND_-CER*	−0.47 (−0.72 to 0.10)	−0.50 (−0.74 to 0.15)	−0.26 (−0.58 to 0.14)	0.29 (0.08–0.48)
Patlak *K_ND_-A*	0.68 (0.40–0.84)	0.73 (0.48–0.87)	0.53 (0.19–0.76)	0.82 (0.74–0.88)
Patlak *K_ND_-B*	−0.04 (−0.42 to 0.35)	−0.14 (−0.50 to 0.26)	−0.13 (−0.49 to 0.26)	0.47 (0.28–0.62)
Patlak *K_ND_-CER-ND*	0.65 (0.36–0.83)	0.63 (0.33–0.82)	0.51 (0.16–0.75)	0.83 (0.75–0.89)
Patlak *K_ND_-A*′	0.72 (0.46–0.86)	0.79 (0.58–0.90)	0.57 (0.24–0.78)	0.84 (0.77–0.90)
Patlak *K_ND_-C*	0.72 (0.46–0.86)	0.78 (0.57–0.90)	0.58 (0.25–0.79)	0.85 (0.77–0.90)
SBI_30–40 min_	0.69 (0.42–0.85)	0.75 (0.51–0.88)	0.59 (0.26–0.79)	0.82 (0.74–0.88)
SBI_50–60 min_	0.69 (0.42–0.85)	0.74 (0.50–0.88)	0.59 (0.26–0.79)	0.82 (0.74–0.88)

**Figure 3.**
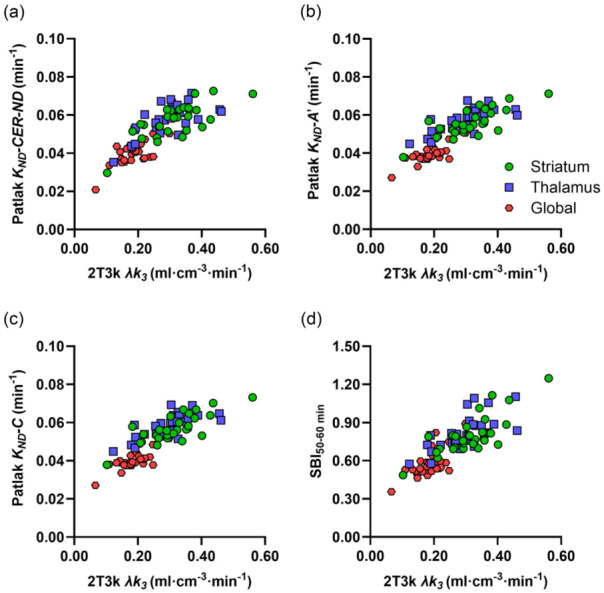
Plasma-input 2T3k λ*k_3_* values in striatum, thalamus and a global cortical region versus *K_ND_* values based on reference Patlak analysis with a subject-specific theoretical CER-ND TAC based on *K_1_* and *k_2_* of the 2T3k model (a), using the modified CER reference TACs according to methods A′ (b) and C (c), and versus a SBI defined as the ratio of target tissue uptake at 50–60 min p.i. to peak CER uptake (d). CER-ND: non-displaceable cerebellum; SBI: specific binding index.

Figure 3 shows the relationship between plasma-input 2T3k λ*k_3_* values and reference Patlak *K_ND_* values based on the subject-specific non-displaceable reference TAC, the modified cerebellar corrections for [^11^C]L-deprenyl-D2 binding A′ and C, as well as SBI_50–60 min_. The corresponding Spearman correlation coefficients are provided in Table 1. As for 2T3k *K_ND_* and method A, moderate correlations were generally found between plasma-input 2T3k λ*k_3_* and reference Patlak *K_ND_* across all VOIs and correction methods. Correlations were higher in the subcortical regions than in the global cortical region. The highest correlation between *K_ND_* obtained with reference tissue models and plasma-input 2T3k λ*k_3_* was observed with correction method C, though only slightly higher than for methods A and A′. As mentioned above, the fit parameters for method C differed significantly between groups, so group-specific reference TAC correction parameters were also tested for this method. This did not improve the overall correlation, which remained identical to that obtained using the median parameter values derived from all subjects (data not shown). Use of the non-displaceable cerebellum TAC as reference curve provided comparable results as methods A, A′ and C. Results obtained using RRTM with various correction methods were nearly identical to those found for the reference Patlak methods (data not shown). As seen in Figure 3(d) and Table 1, SBI shows a similar correlation to 2T3k λ*k_3_* as reference Patlak *K_ND_* using the best-performing cerebellar correction methods (A′ and C). The correlation between SBI and plasma-input 2T3k λ*k_3_* was identical for both time intervals: 30–40 and 50–60 min.

Supplementary Figure S1 shows that the relations between the plasma-input 2T3k *K_ND_* values and non-invasive Patlak *K_ND_* for the non-displaceable cerebellum reference curve and the different correction methods, as well as between SBI_50–60 min_ and 2T3k *K_ND_*, are similar with the exception of method B. Reference tissue corrections A′ and C show moderate correlations with slopes close to unity, and A shows this to a lesser extent. Reference Patlak *K_ND_* based on method B correlates poorly with 2T3k *K_ND_*. Spearman correlations and Deming regression slopes are given in Supplementary Table S1.

**Figure 4.**
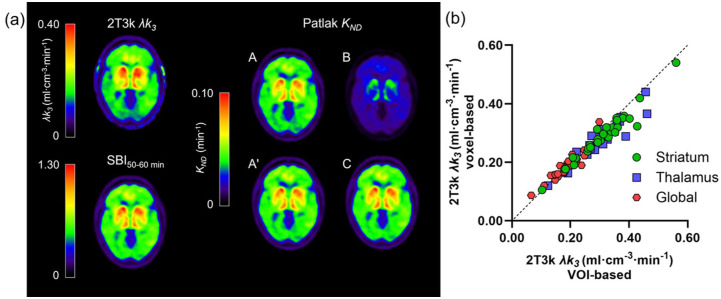
(a) Parametric [^11^C]L-deprenyl-D2 images for a representative subject with SPMS (45 years, female). The top left shows the plasma-input 2T3k λ*k_3_* image and the bottom left the SBI_50–60 min_ image. On the right, the reference Patlak *K_ND_* images generated using the four different cerebellar correction methods are shown. (b) Relation between voxel-based and VOI-based 2T3k λ*k_3_*. The dashed line represents the line of identity.

Figure 4(a) shows parametric [^11^C]L-deprenyl-D2 λ*k_3_*, reference Patlak *K_ND_* with different cerebellar correction methods, and SBI_50–60 min_ images for a representative subject with SPMS. Visually, the SBI and *K_ND_* images show a similar pattern to the λ*k_3_* image, albeit with cerebellar correction method B resulting in an underestimation of *K_ND_* compared to the other methods, as expected based on Figure 2(d). As shown in Figure 4(b), VOI-based and voxel-based 2T3k λ*k_3_* values demonstrated consistently high correlation and agreement. Correlation was particularly strong in the striatum (ρ = 0.96; 95% CI 0.90–0.98, slope = 0.90; 95% CI 0.78–1.02), and remained high in the thalamus (ρ = 0.92; 95% CI 0.83–0.96, slope = 0.86; 95% CI 0.71–1.01) and global cortical region (ρ = 0.92; 95% CI 0.83–0.96, slope = 0.96; 95% CI 0.61–1.30). These findings reflect an overall strong correlation across all regions (ρ = 0.97; 95% CI 0.95–0.98, slope = 0.86; 95% CI 0.80–0.93). Very high correlation and agreement were also observed between VOI-based and voxel-based reference Patlak *K_ND_* values, with Spearman correlation coefficients and slopes close to unity.

**Figure 5.**
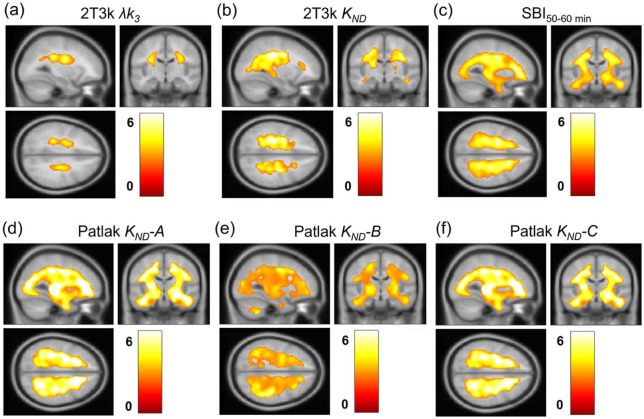
Voxel-wise comparison between patients with SPMS and healthy controls. *T*-score maps display clusters with significantly higher 2T3k λ*k_3_* and *K_ND_* in (a) and (b), and SBI_50–60 min_ values in (c), indicating increased MAO-B activity in SPMS patients. Reference Patlak *K_ND_* values with cerebellar correction A, B and C are shown in (d)–(f).

Voxel-wise comparisons between SPMS patients and healthy controls based on the different models are presented in Figure 5. Both invasive and non-invasive methods showed a significant increase in MAO-B activity in white matter in SPMS patients compared to healthy controls. However, the reference Patlak methods and SBI images overestimated the extent of the difference between SPMS patients and healthy controls. No significant differences were found between the AHSCT group and healthy controls for plasma-input 2T3k λ*k_3_* values. However, significant differences were observed in *K_ND_* values with the modified reference Patlak methods (data not shown).

Table 2 shows the total volumes of the areas with increased MAO-B activity seen in Figure 5, along with mean and maximum significance levels, quantitatively illustrating the overestimation observed with the reference methods compared to plasma-input 2T3k λ*k_3_*. The reference Patlak methods consistently yielded volume estimates more than 10 times larger than those found with plasma-input 2T3k λ*k_3_* for regions exceeding the significance threshold (*t* = 2.55, *p* < 0.01), along with elevated *t*-values within these clusters.

**Table 2.** Volume of *t*-map clusters presented for plasma-input 2T3k λ*k_3_* and *K_ND_* estimates, as well as for SBI_50–60 min_ and the different reference Patlak methods, along with their corresponding mean and maximum *t*-values within these clusters.

Model	Volume (cm^3^)	Mean *t*-value	Max *t*-value
2T3k λ*k_3_*	32	3.12	5.23
2T3k *K_ND_*	155	3.47	7.06
SBI_50–60 min_	381	3.46	6.48
Patlak *K_ND-A_*	527	4.31	9.94
Patlak *K_ND-B_*	454	3.20	5.66
Patlak *K_ND-C_*	483	4.15	9.45

The results of the simulations are shown in Supplementary Figure S2. Fitted 2T3k *K_ND_* values showed a high correlation and agreement with the true values (ρ = 0.99, slope 1.01), indicating high accuracy and precision of 2T3k *K_ND_* for noise levels corresponding to those at the VOI level. When assuming identical *V_ND_* in target and reference tissue, reference Patlak *K_ND_* also correlated and agreed well with 2T3k *K_ND_* (ρ = 1.00, slope 1.02). However, when inducing a variation between target and reference tissue *V_ND_* comparable to that found in the clinical data, the correlation between reference Patlak *K_ND_* and 2T3k *K_ND_* decreased to comparable levels as those seen in Supplementary Figure S1 (ρ = 0.87, slope 1.14), which explains the poor to moderate correlations between reference tissue and plasma-input models found in this work.

## Discussion

In this study, we demonstrate that the implementation of parametric 2T3k λ*k_3_* images with arterial input function using the basis function method agreed well with VOI-based weighted nonlinear regression analysis. Non-invasive methods showed moderate overall correlation with plasma-input λ*k_3_* for the best-performing modified reference and semi-quantitative methods, with lower correlations for individual VOIs, such as the striatum, thalamus and a global cortical region, indicating that they are not a robust alternative to quantifying MAO-B activity using [^11^C]L-deprenyl-D2. To the best of our knowledge, neither the parametric nor non-invasive methods have previously been thoroughly validated for [^11^C]L-deprenyl-D2.

The net uptake rate relative to the non-displaceable volume of interest *K_ND_*, even when based on arterial input data, did not show a high correlation with λ*k_3_* (Figure 2(a)), in the global cortical but especially in the subcortical regions with high levels of MAO-B. This in itself is not surprising, as λ*k_3_* and *K_ND_* are different measures of tracer binding with different relationships to the irreversible binding rate constant *k_3_*. λ*k_3_* is proportional to *k_3_* provided *V_ND_* (= λ) is constant, whereas *K_ND_* is proportional to *k_3_* if *k_3_* ≪ *k_2_*, which is somewhat fulfilled for [^11^C]L-deprenyl-D2, with a decreasing slope in the relationship between *K_ND_* and *k_3_* for increasing *k_3_* values relative to *k_2_*. However, as λ*k_3_* was previously suggested as the preferred measure of [^11^C]L-deprenyl-D2 binding, we chose to use this as the gold standard value in the present work.

The two previously suggested non-invasive quantification methods, accounting for MAO-B binding in the cerebellar TAC either by multiplication with an exponential function (A)^16^ or by convolution subtraction of an exponential function (B),^1^ did not provide *K_ND_* values that correlated well with λ*k_3_* either, but also not with 2T3k *K_ND_* (Supplementary Figure S1). Efforts to optimise the previously proposed method A (equation (1)) by basing its parameters on a fit to the theoretical non-displaceable reference TAC (A′), or use of a sigmoid function to improve the reference TAC’s similarity to the theoretical non-displaceable reference TAC (C) (Figure 1), did not result in much improved agreement with the plasma-input model. For methods A′ and C, we used the median values of the fitted parameters from all subjects. Although we found a significant difference in parameters for method C between subject groups, the use of group-specific parameters did not improve overall correlation compared to using the median parameters derived from all subjects. However, our aim was to establish correction methods with parameters applicable to any subject, regardless of disease status, and implementing corrections tailored to specific patient groups would defeat this purpose.

The convolution subtraction correction method B (equation (3)) exhibited a very weak and negative correlation between *K_ND_* and λ*k_3_*. Considering the shape of the reference curve estimated by this method, as seen in Figure 1(b), which does not correspond to the expected TAC for a region with only non-displaceable binding, this is not surprising. As mentioned in the introduction, this method has been previously used in a number of studies of neurodegenerative diseases.^11–14,31–33^ However, based on the present results, *K_ND_* based on method B does not reflect MAO-B binding. Hence, it remains to be investigated how the results of those studies should be interpreted if they do not reflect MAO-B activity.

Even the use of the subject-specific theoretical non-displaceable cerebellum curve itself as reference TAC did not result in a strong correlation with 2T3k *K_ND_*. One explanation for this could be that the non-displaceable cerebellum curve is not well defined, but the simulations showed high accuracy and precision of not only 2T3k *K_ND_* but also of *k_2_* and *k_3_* themselves (data not shown). This is further supported by a test-retest study, which demonstrated that *k_3_* has similar reproducibility to λ*k_3_* in healthy subjects.^45^ However, *k*_*3*_ is not a valid option for parametric images, as the noise level of individual voxel TACs prohibits robust estimation of *k*_*3*_. Nevertheless, the simulations show that the moderate correlation of Patlak *K_ND_*, using the non-displaceable cerebellum TAC as reference, with 2T3k *K_ND_* is likely due to differences in *V_ND_* between target and reference tissues rather than the robustness of 2T3k *K_ND_*. Hence, even the existence of a reference region completely devoid of specific binding would not have resulted in high correlations between non-invasive *K_ND_* and 2T3k *K_ND_* in the presence of differences in *V_ND_* between target and reference tissues as observed in the clinical data. This underscores the difficulty of achieving a fully reliable non-invasive alternative, as irreversible reference tissue models seem particularly sensitive to differences in *V_ND_* between target and reference tissues.

Although correlations between *K_ND_* and λ*k_3_* are moderate at best using the different approaches for correction of the cerebellar TAC, the non-invasive *K_ND_* images as well as SBI do visually resemble the λ*k_3_* image as shown in Figure 4(a). However, as presented in Table 2, *K_ND_* parametric images and SBI overestimate both the extent and magnitude of the significant difference between SPMS patients and healthy controls at the voxel level (Figure 5). Still, the visual resemblance between non-invasive parametric images and λ*k_3_* images suggests that, although absolute quantification is challenging, they may be useful for assessment of intra-individual qualitative changes in MAO-B. If non-invasive methods are to be used, SBI provides a simple, easily implemented quantitatively comparable alternative to the best-performing modified reference tissue methods, while also enabling shorter scan duration if the 30–40 min interval is used.

In conclusion, parametric images of λ*k_3_* obtained using a basis function implementation of the plasma-input irreversible two-tissue compartment model showed good agreement with the results from VOI-based nonlinear regression analysis. Full kinetic analysis with arterial input function is required for accurate assessment of MAO-B activity with [^11^C]L-deprenyl-D2. Finally, this study highlights the importance of thorough validation of simplified methods prior to their use in clinical studies.

## Supplemental Material

sj-docx-1-jcb-10.1177_0271678X251384264 – Supplemental material for Non-invasive modelling and parametric methods for quantification of MAO-B activity using [11C]L-deprenyl-D2 PET Supplemental material, sj-docx-1-jcb-10.1177_0271678X251384264 for Non-invasive modelling and parametric methods for quantification of MAO-B activity using [11C]L-deprenyl-D2 PET by Karolina Hedman, My Jonasson, Lieuwe Appel, Andreas Tolf, Joachim Burman and Mark Lubberink in Journal of Cerebral Blood Flow & Metabolism

sj-docx-2-jcb-10.1177_0271678X251384264 – Supplemental material for Non-invasive modelling and parametric methods for quantification of MAO-B activity using [11C]L-deprenyl-D2 PET Supplemental material, sj-docx-2-jcb-10.1177_0271678X251384264 for Non-invasive modelling and parametric methods for quantification of MAO-B activity using [11C]L-deprenyl-D2 PET by Karolina Hedman, My Jonasson, Lieuwe Appel, Andreas Tolf, Joachim Burman and Mark Lubberink in Journal of Cerebral Blood Flow & Metabolism
