# Supplementary material for: Non-invasive modelling and parametric methods for quantification of MAO-B activity using [11C]L-deprenyl-D2 PET
Source: J Cereb Blood Flow Metab. 2026 Jan 16:0271678X251384264. Online ahead of print. doi: 10.1177/0271678X251384264 (PMC12812065; doi:10.1177/0271678X251384264)
Supplement: sj-docx-1-jcb-10.1177_0271678X251384264 – Supplemental material for Non-invasive modelling and parametric methods for quantification of MAO-B activity using [11C]L-deprenyl-D2 PET [file sj-docx-1-jcb-10.1177_0271678X251384264.docx]

**Figure S1.** Plasma-input 2T3k *K_ND_* versus non-invasive *K_ND_* based on a non-displaceable cerebellum reference TAC (a), previously published cerebellar TAC corrections A (b) and B (c), and modified cerebellar TAC corrections A' (d) and C (e). Panel (f) shows SBI_50-60 min_ versus 2T3k *K_ND_*.

**Table S1.** Spearman correlation (*ρ*) and Deming regression between plasma-input 2T3k *K_ND_* and reference Patlak *K_ND_* estimates using different cerebellar TAC correction methods. Data in parentheses represent 95% confidence intervals.

**Figure S2.** Simulation results: (a) fitted 2T3k *K_ND_* versus simulated *K_ND_*; (b) fitted Patlak *K_ND_* using a true reference tissue (*V_ND_* equal in target and reference tissues) versus true *K_ND_*; (c) fitted Patlak *K_ND_* using a reference tissue with different *V_ND_* in target and reference tissues (*K_1_* and *k_2_* in reference tissue varied with ±15%).
