# Supplementary material for: Non-invasive modelling and parametric methods for quantification of MAO-B activity using [11C]L-deprenyl-D2 PET
Source: J Cereb Blood Flow Metab. 2026 Jan 16:0271678X251384264. Online ahead of print. doi: 10.1177/0271678X251384264 (PMC12812065; doi:10.1177/0271678X251384264)
Supplement: sj-docx-2-jcb-10.1177_0271678X251384264 – Supplemental material for Non-invasive modelling and parametric methods for quantification of MAO-B activity using [11C]L-deprenyl-D2 PET [file sj-docx-2-jcb-10.1177_0271678X251384264.docx]

**Table S1.** Spearman correlation (*ρ*) and Deming regression between plasma-input 2T3k *K_ND_* and reference Patlak *K_ND_* estimates using different cerebellar TAC correction methods. Data in parentheses represent 95% confidence intervals.

| Model | Striatum | Thalamus | Global | Overall | Slope |
| --- | --- | --- | --- | --- | --- |
| Patlak *K_ND_-CER-ND* | 0.47  (0.10 – 0.72) | 0.14  (-0.26 – 0.50) | 0.64  (0.33 – 0.82) | 0.70  (0.56 – 0.80) | 1.11  (0.90 – 1.31) |
| Patlak *K_ND_-A* | 0.46  (0.10 – 0.72) | 0.17  (-0.23 – 0.52) | 0.50  (0.14 – 0.74) | 0.72  (0.59 – 0.81) | 0.86  (0.69 – 1.03) |
| Patlak *K_ND_-B* | 0.10  (-0.30 – 0.46) | -0.50  (-0.74 – (-0.14)) | -0.19  (-0.53 – 0.21) | 0.47  (0.28 – 0.63) | 0.24  (0.15 – 0.33) |
| Patlak *K_ND_-A'* | 0.48  (0.12 – 0.73) | 0.28  (-0.12 – 0.60) | 0.54  (0.20 – 0.76) | 0.73  (0.61 – 0.82) | 0.93  (0.76 – 1.11) |
| Patlak *K_ND_-C* | 0.48  (0.12 – 0.73) | 0.28  (-0.11 – 0.60) | 0.56  (0.22 – 0.77) | 0.73  (0.61 – 0.82) | 0.97  (0.79 – 1.15) |
